# Underwater Photogrammetry and Object Modeling: A Case Study of Xlendi Wreck in Malta

**DOI:** 10.3390/s151229802

**Published:** 2015-12-04

**Authors:** Pierre Drap, Djamal Merad, Bilal Hijazi, Lamia Gaoua, Mohamad Motasem Nawaf, Mauro Saccone, Bertrand Chemisky, Julien Seinturier, Jean-Christophe Sourisseau, Timmy Gambin, Filipe Castro

**Affiliations:** 1 Aix Marseille Université, CNRS, ENSAM, Université De Toulon, LSIS UMR 7296,13397 Marseille, France; Djama.Merad@univ-amu.fr (D.M.); Bilal.Hijazi@univ-amu.fr (B.H.); Lamia.Gaoua@univ-amu.fr (L.G.); Mohamad-Motasem.Nawaf@univ-amu.fr (M.M.N.); Mauro.Saccone@uniroma3.it (M.S.); 2 COMEX, COmpanie Maritime d’EXpertise 36 boulevard des Océans, 13009 Marseille, France; b.chemisky@comex.fr (B.C.); j.seinturier@comex.fr (J.S.); 3 Aix Marseille Université, CNRS, Ministère de la Culture et de la Communication, CCJ UMR 7299, 13094 Aix En Provence, France; Jean-Christophe.Sourisseau@univ-amu.fr; 4 Archaeology Centre (Car Park 6), University of Malta, Msida MSD 2080, Malta; Timmy.Gambin@um.edu.mt; 5 Ship Reconstruction Laboratory 4352 TAMU, Texas A & M University, College Station, TX 77843, USA; fvcastro@neo.tamu.edu

**Keywords:** underwater photogrammetry, visual odometry, ontologies, underwater archaeology

## Abstract

In this paper we present a photogrammetry-based approach for deep-sea underwater surveys conducted from a submarine and guided by knowledge-representation combined with a logical approach (ontology). Two major issues are discussed in this paper. The first concerns deep-sea surveys using photogrammetry from a submarine. Here the goal was to obtain a set of images that completely covered the selected site. Subsequently and based on these images, a low-resolution 3D model is obtained in real-time, followed by a very high-resolution model produced back in the laboratory. The second issue involves the extraction of known artefacts present on the site. This aspect of the research is based on an *a priori* representation of the knowledge involved using systematic reasoning. Two parallel processes were developed to represent the photogrammetric process used for surveying as well as for identifying archaeological artefacts visible on the sea floor. Mapping involved the use of the CIDOC-CRM system (International Committee for Documentation (CIDOC)—Conceptual Reference Model)—This is a system that has been previously utilised to in the heritage sector and is largely available to the established scientific community. The proposed theoretical representation is based on procedural attachment; moreover, a strong link is maintained between the ontological description of the modelled concepts and the Java programming language which permitted 3D structure estimation and modelling based on a set of oriented images. A very recently discovered shipwreck acted as a testing ground for this project; the Xelendi Phoenician shipwreck, found off the Maltese coast, is probably the oldest known shipwreck in the western Mediterranean. The approach presented in this paper was developed in the scope of the GROPLAN project (Généralisation du Relevé, avec Ontologies et Photogrammétrie, pour l'Archéologie Navale et Sous-marine). Financed by the French National Research Agency (ANR) for four years, this project associates two French research laboratories, an industrial partner, the University of Malta, and Texas A & M University.

## 1. Introduction

At the convergence of computer science and the humanities, the Ontology and Photogrammetry; Generalizing Surveys in Underwater and Nautical Archaeology (GROPLAN) project, brings together researchers from academia and industry. The main fields of activity of the GROPLAN project are in underwater and nautical archaeology.

Our central objective is to build an information system based on ontologies. In turn, such an information system will provide a formal framework as well as tools to express and manage digital content and expert knowledge in a homogenous manner. Our second objective is the development of methods for collecting data from sites and the integration of such data into the information system during the acquisition phase. In this paper these two aspects are addressed in the context of the exploration of an exceptional archaeological site—The Phoenician shipwreck off Gozo Xlendi. Resting at a depth of 110 m, it is probably the oldest ancient shipwreck in the central Mediterranean.

The photogrammetric survey was based on an original approach to underwater photogrammetry with scientific assets provided by a partner in the GROPLAN project. A photogrammetric process and the corpus of surveyed objects were combined for ontological formalization. Our approach is based on procedural attachment with the ontology perceived as a combination of the Java class structure. In turn, this manages the photogrammetric survey and the measurement of artefacts. This allows the establishment of reasoning for the ontologies as well as intensive calculations using Java with the same interface. For the sake of transversality, the ontology used to describe the archaeological artefacts from a measurement point of view can be seen as an extension of the CIDOC-CRM ontology used for museo-graphical objects [1,2].

We will also be initiating a process to facilitate the generalisation of this approach to make it available in the field of nautical archaeology. Such an aim will be achieved through several site-specific case studies. The measurement/knowledge relationship will be studied in the scope of nautical archaeology in collaboration with Texas A & M University which has already started working on formalizing ship structures. For a number of years, Texas A & M has also conducted various underwater archaeological excavations in the Mediterranean.

The resolutely interdisciplinary aspect of the GROPLAN project is reflected in the diversity of its partners as well as in the complementary nature of their activities; a computer science lab with an extensively experienced team in close-range photogrammetry, three archaeology departments and two private companies. One of the companies specialises in underwater exploration whereas the other focuses on dimensional control. The key people in this project are computer scientists, photogrammetrists, archaeologists, anthropologists, engineers, and oceanographers. The vast geographic scope of the project, which includes France, Malta and Texas (USA) also highlight GROPLAN’s diversity.

### 1.1. Context

This project deals with the process and the problems of archaeological survey from the perspective of knowledge through the use of photogrammetry. Recent developments in computer vision and photogrammetry make this latter technique a near ideal tool. It could actually be deemed as an essential tool for archaeological survey. Indeed, photogrammetry provides an easy setup remote sensing technique with low implementation costs. This technique also provides denser 3D models when compared to those obtained using laser scanners at ground-level conditions (modern laser scanners can only provide up to 100 K depth points per measuring cycle). In the context of underwater archaeology it is undeniably a must because there is no real alternative.

The main idea of this project is based on the fact that survey, which takes place in the scope of nautical and underwater archaeology relies on a complex well-established methodologies that have evolved over time. The notion of a model as a formalisation of archaeological knowledge is used to guide the survey.

The confluence between the ever-increasing quantities of measured data and knowledge that is progressively formalized (ontologies, semantic networks) raises the issue of the development and automation of survey systems that are able to make the most out of the confrontation of these two aspects.

Measurement data is decoupled from the resulting geometry. On the one hand, the process records all acquired data (measurements, graphical data, annotations, archaeological data or that pertaining to the field of study). On the other hand, after the computation and validation phases of these data the resulting graphical (2D or 3D) images represent the result of a query into the ontologies that manage all collected information.

The measurements obtained through the photogrammetry survey are sparse and these partially fit the theoretical model. A fusion scheme allows the integration between measurements and model. Once instantiated this will produce a graphical representation that is reflective of the needs as expressed by the end-user.

This project is still underway and its first phase focuses on the issues of underwater archaeology through an approach dedicated to a corpus of amphorae. Experiments in underwater archaeology took place on the Phoenician shipwreck in Malta done in collaboration with the University of Malta. The Phoenician shipwreck represents an exceptional site from various points of view. Recently discovered through systematic exploration of the seabed, this deep-water shipwreck lies at a depth of approximately 110 m. From a logistical point of view it is located in a very favourable area on a sandy plateau relatively close to the shore and free of all marine vegetation. The earliest survey available brought to light the upper part of a well-preserved cargo with minimal disturbance. It consists of amphorae, urns and large millstones that explicitly evoke the shape of the ship which measured approximately 12–14 m long and 4–5 m wide. Results from a sub-bottom-profiler survey suggest the existence of a second layer of amphorae. This was confirmed through the photogrammetrical survey performed in July 2014 as part of this project. The first sonar images also confirm this hypotheses and clearly show that the boat and its cargo landed on the seabed before sand rapidly covered the site, thus protecting it from further erosion. The shipwreck is in itself exceptional. Firstly, due to its configuration and its state of preservation which combines to make it particularly well-suited for our experimental 3D modelling project. Exploration of the first layer of amphorae reveals a mixed cargo consisting of items from both Western Phoenicia and the Tyrrhenian-area, which both match the period ranging from between the end of the VIII and the first half of the VII centuries BC. This makes it the oldest known wreck in the western Mediterranean and contemporary to the early days of Carthage and the first Greek settlements in the West. The historical importance of this wreck is highlighted by our work. It is the first time that such technologically advanced techniques have been used on this site. Our fieldwork created a real added-value both in terms of innovation and the international reputation of the project itself.

### 1.2. Underwater Photogrammetry and Survey

Deep-water shipwrecks have not been widely tackled by researchers, mainly due to a lack of information as well as issues related to accessibility. The lack of information arises because diving beyond 50 m using standard SCUBA equipment with compressed air is prohibited. The depth limit for divers breathing air is specified by several organisations including BSAC in the UK [3] or French law [4]. Diving beyond this limit requires further in-depth training, the use of enriched air and significant facilities on the surface. Furthermore, these deep-sea wrecks are also protected by various natural physio-chemical factors including low light, cooler temperatures and reduced oxygen. Such factors combine to help further preserve such wrecks.

However, threats to deep-water sites are increasing. One major threats stems new forms of trawling that destroy the surface layer of these sites and interfere with their readability. In fact, the protection that has always been afforded to such sites is now something of the past. Trawling nets today can be deployed to depths of up to 1000 m. Consequently, many of these shipwrecks are presently more likely to be damaged before they can be studied, or even observed.

Aside from very limited accessibility by divers, a deep water site cannot be physically reached by the majority of underwater archaeologists, marine biologists or other experts in related marine sciences. It is therefore important, even crucial, to implement better techniques, which are easily deployed and that are able to accurately survey deep-water sites. This represents one of the interests of our research.

The acquisition system used for the photogrammetric survey was installed on the Rémora 2000 submarine made by COMEX. This two-person submarine has a depth limit of 610 m with a maximum dive time of 5 h. Five hours provides more than enough time for the data acquisition phase of the photogrammetry survey. What is of crucial importance to us are the three high-resolution cameras that are synchronized and controlled by a computer. All three cameras are mounted on a bar located on the submarine just in front of the pilot. Continuous lighting of the seabed is provided by a Hydrargyrum medium-arc iodide lamp (HMI) powered by the submarine. The continuous light is more convenient for both the pilot and the archaeologist who can better observe the site from the submarine. The high frequency acquisition frame rate of the cameras ensures full coverage whereas the large scale of acquired images gives the eventual 3D models extreme precision (up to 0.005 mm/pixel for an ortho-photograph).

Deployed in this way the acquisition system entails zero contact with the archaeological site making it both non-destructive and extremely accurate. The on-board processing within the submarine permits the creation of real-time 3D structure estimation of the area covered by the vehicle. This ensures that the pilot can obtain complete coverage of the survey area before the he returns the vehicle to the surface.

Photogrammetry in an underwater context makes it possible to obtain a comprehensive survey of all visible parts of the site without impacting the objects. Moreover, such a task can be accomplished in relatively short time and with a high degree of precision. This approach offers specialists and members of the general public a complete view of a site that is normally not possible due to the turbidity of the marine environment and a lack of light [5]. This aspect of the survey is described in the second section of this paper when discussing the example of the Phoenician shipwreck off Malta.

Our initial focus shall be on an aspect that is often neglected in discussions related to survey techniques: The relationship with knowledge. As archaeological excavations often result in irreversible damage, it is important to ensure that they are accompanied by relevant documentation. Such documentation must take into account the accumulated knowledge gained from the site. This documentation is generally iconographic and textual. Graphic representations of archaeological sites such as drawings, sketches, photographs, topographic renditions, artist impressions and photogrammetric studies are all essential phases or archaeological surveys. However, as highlighted by Olivier Buchsenschutz in his introduction to the conference entitled “Images and archaeological surveys—From proof to demonstration”, held in Arles in 2007 [6]: “Even when very accurate, drawings only retain certain observations to support a demonstration, just as a speech only retains certain arguments, but this selection is not generally explicit”. In a certain way, this sets the foundation for the further development of this work: Surveys are both a metric representation of the site as well as an interpretation of the same site by the archaeologist.

Surveys are very important components of the documentation and their importance is mostly due to the fact that concepts handled by archaeologists during an excavation are strongly related to space. The very structure of an excavation is based around the notion of a stratigraphic unit. Inherited from a geological approach and subsequently formalised for archaeology by E.-C. Harris [7]. Stratigraphic units are connected to each other through geometrical, topological and temporal relationships and give a structure to the reading of the excavation.

Two families of objects must be distinguished: parts of terrain, or more generally, areas of space that are organised into stratigraphic units and the artefacts that must be positioned in that space. Such an exercise is essential for post-excavation studies that are conducted after the site has been “destroyed”. In this paper, we will principally cover the second of the aforementioned objects: artefacts. The survey is therefore based on a priori knowledge, formalized in close collaboration with archaeologists. This knowledge was used during the measurement phase and communicated right through the final representations of the site. Based principally on our knowledge of measured artefacts, this approach used this knowledge to measure the size and localise the object.

Finally, it is imperative to note that archaeological data is, by its very nature, incomplete; mainly because it is heterogeneous and discontinuous as well as being subject to possible updates and revisions (both theoretical and technological). The verification of the final coherence of the survey will be one of the primary objectives of this work.

### 1.3. Archaeological Experimentation

The shipwreck was first located by Aurora Trust in 2008 during a systematic survey off the coasts of Malta and Gozo that it was conducting with state of the art digital side scan sonar. This ongoing broad survey is authorised by the Superintendence of Cultural Heritage with the aim of creating an inventory of all the underwater ruins located in Malta’s territorial waters at depths ranging from 50 to 150 m. Further studies of one sonar target by a remote operated vehicle helped identify the site as a very ancient shipwreck. A collaborative group was gradually created by Timmy Gambin in order to put together a strategy adapted for an in-depth the study of this shipwreck.

**Figure 1:**
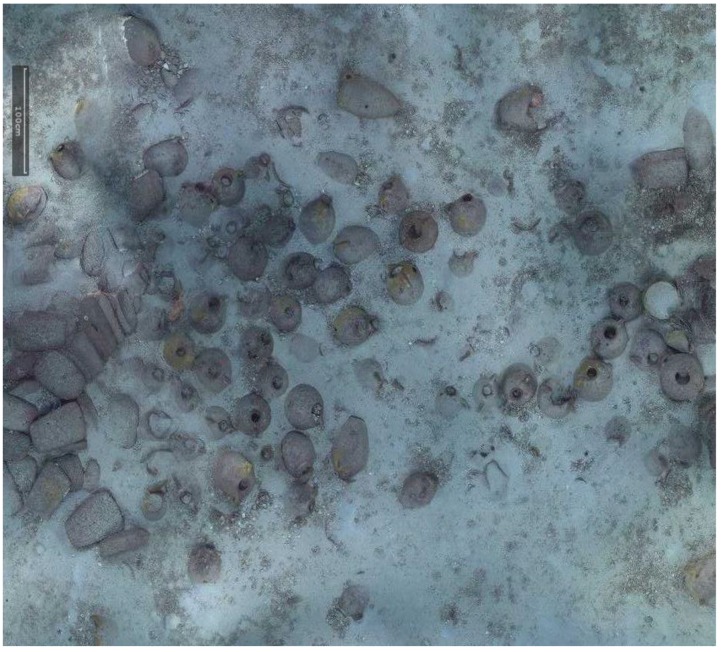
A snapshot taken from a very high resolution orthophoto, the full resolution image can be found in the project’s website (http://www.groplan.eu). The overall image resolution is 41,507 × 60,377 pixels at a scale of 0.005 mm per pixel.

The shipwreck is located near a stretch of coastline that is characterised by limestone cliffs that plunge into the sea and whose foundation rests on a continental shelf at an average depth of 100 m below sea level. The shipwreck rests on a practically flat area of this submerged plateau at a depth of 110 m. Sonar images as well as the image of the entire archaeological deposit shows that the cargo remains tightly grouped together and appears to have been only slightly disturbed. An “amphora mound” effect could not be detected indicating the good state of preservation of the transported goods. It is generally accepted that amphora-mounds arise when the hull disintegrates in open water (after sinking) due to the absence of sedimentation processes. This type of phenomenon results in a haphazard spilling and spreading out of cargo. With the site currently under discussion the ship’s longitudinal axis and cargo remain visible, spread out over 12 m and easily identifiable. The maximum width of the non-disturbed part is approximately 5 m (see orthophotograph in Figure 1).

**Figure 2:**
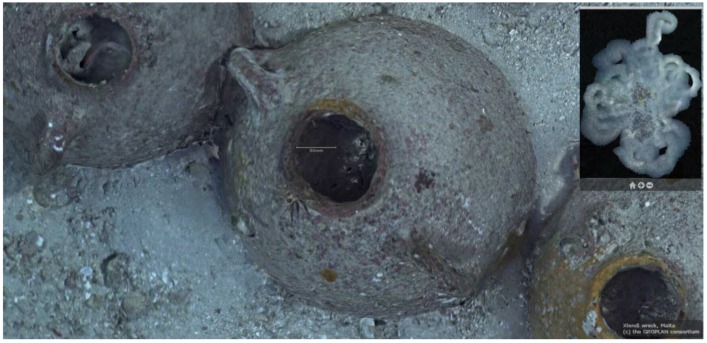
Very high resolution orthophoto from the project’s website (http://www.groplan.eu). A zoomed-in of the image shown in Figure 1.

Some matters arising from this project may, at first glance, seem distant from the primary interest usually afforded by archaeologists to underwater shipwrecks. However, initial discussions on potential survey strategies brought to light questions involving new requirements. These requirements were progressively established through cross-disciplinary dialogue between collaborators. For the archaeologist, the production of reliable documents from geo-referenced ortho-photographic surveys such as those described here (see Figure 1 and Figure 2 below) provides significant added-value. However, it became apparent that what may seem to be an accomplishment enabling the interpretation of the wreck’s visible surface layer proved in practice to be insufficient.

Given that the site being explored is as fragile as it is exceptional, as are other deep water sites, it was imperative that past mistakes, such as haphazard approaches to survey work, be avoided. Preliminary reflections identified two priority actions: (1) the detection, localisation and mapping of deposits; and (2) their characterisation in terms of nature, contents, organisation and time-frame. Thus, the cultural and scientific tasks were closely intertwined. However, a degree of experimentation with available tools was still necessary. This is because such an approach would be needed to quickly provide the desired information despite the constraints that arise from working in a deep-water environment. This is reflective of GROPLAN project’s spirit, which seeks developments in the fields of photogrammetry and shape analysis through ontology. In turn, such developments are immediately deployed in the field of archaeological research, management and protection of submerged cultural assets.

Contrary to other archaeological deposits located at depths of less than 50 m, which are often eroded and greatly pillaged, deep-sea wrecks are generally better preserved and offer the chance for a more contextual and spatial approach. As such, the Phoenician wreck offers an interesting and promising site for scientific application and experimentation. This is due to its aforementioned excellent preservation. Its excellent state of preservation can be further deduced through the observation of precisely grouped divisions of located objects as well as their variety. Preliminary observations enabled a precursory characterisation of the objects that were identified using available traditional typological tools. Based on these “traditional” observations, standard in archaeological practice, the groups of objects were identified and tentatively dated. The main idea here is to go (this already established) to an automatic shape recognition experiment. The latter starting from a photogrammetric survey based on the ontological analysis of amphora shapes. The interest of conducting such an experiment is to build a tool that can automatically recognise objects, even if only partially visible, by verifying the relevance of its responses all along the gradual construction of the descriptive arguments as well as by comparing it to an archaeological analysis. The aim is to build a descriptive tree structure that is sufficiently accurate so that a selected shape can be automatically recognised from a repository of known shapes and clearly identified beforehand.

**Figure 3:**
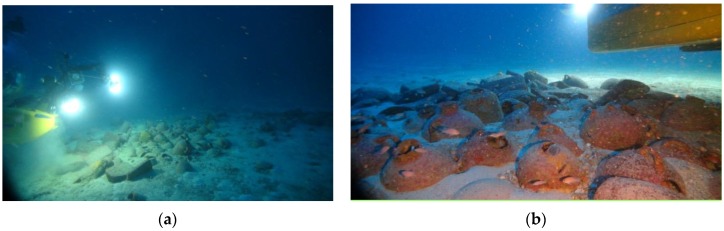
Examples of the wreck images taken by the ROV Super-Achille deployed during the photogrammetric survey carried out from the submarine Rémora 2000. The mixed” cargo is visible on both (**a**) and (**b**) images.

Due to its “mixed” cargo (see Figure 3), which consists of different types of objects, the Phoenician wreck is particularly well suited for this type of approach. In the medium term, once the library of shapes is sufficiently populated, such an approach should permit automatic explorations at greater depths, offering a preliminary reasoned identification of objects from the cargo of deep-sea wrecks, both rapidly and with few resources.

## 2. Mission Field, Underwater Survey

### 2.1. The Survey Approach

The photogrammetric system was originally designed to be mounted on a lightweight remotely controlled vehicle, implying an optimised size and weight for the assembly—The system is called ROV-3D [3].

A previous version, presented here, was mounted on the submarine Remora 2000. In the ROV 3D configuration, the operator has direct access to the on-board acquisition unit (UAE) via a high-speed Ethernet link between the inside of the inhabited vehicle and the system on the surface. In this version, a connection was established between the embedded sub system, which was fixed on the submarine and the pilot (inside the submarine).

To facilitate the portability, all the components of the photogrammetric system were arranged in a removable assembly that could be attached to the submarine technical bar or under the remotely controlled vehicle (ROV).

Our setup implements a synchronized acquisition of high and low resolution images by video cameras forming a trifocal system. The three cameras are independently mounted in separate waterproof housings. This implies two separate calibration phases: The first one is carried out on each camera housing in order to compute intrinsic parameters and the second one is done to determine the relative position of the three cameras which are securely mounted on the rigid platform. The second calibration can easily be done before each mission. This calibration phase affects the final 3D model scale. This trifocal system is composed of one high-resolution, full-frame camera synchronized at 2 Hz and two low-resolution cameras synchronized at 10 Hz (see Table 1).

**Table 1:** Intrinsic parameters for the trifocal system.

	Cam 1, Low Resolution	Cam 2, Low Resolution	Cam 3, High Resolution
Manufacturer	Allied Vision Technologies		
Model	AVT PROSILICA GT1920		AVT PROSILICA GT6600
Focal length (mm)	5.7578		28.72
Sensor size (mm)	6.61 × 8.789		24 × 36
Image resolution (px)	1456 × 1936		4384 × 6576

The lighting, which is a crucial part in photogrammetry, must meet two criteria: the homogeneity of exposure for each image and consistency between images. This is why we use the HMI light system mentioned earlier.

**Figure 4:**
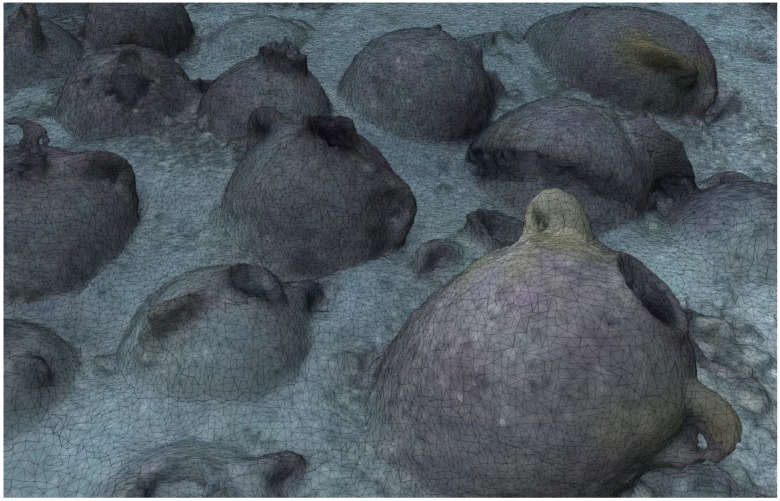
A snapshot of the 3D survey at low resolution for web display through *skechfab*, available on the GROPLAN website (http://www.groplan.eu).

The trifocal system has two different aims: the first one is the real-time computation of system pose and the 3D reconstruction of the zone of seabed visible from the cameras. The operator can pilot the submarine using a dedicated application that displays the position of the vehicle in real-time. A remote video connection also enables the operator to see the images captured by the cameras in real-time. Using available data, the operator can assist the pilot to ensure the complete coverage of the zone to be surveyed. The pose is estimated based on the movement of the vehicle between two consecutive frames. To do this, homologous points are found on two successive pairs of images in low resolution (four images) and sent to the surface computer. The computation is described in detail later in this paper. The second goal is to perform an offline 3D reconstruction of a high resolution metric model. This process involves the use of the high-resolution images for the production of a dense model (see Figure 4) that is scaled based on baseline distances.

**Figure 5:**
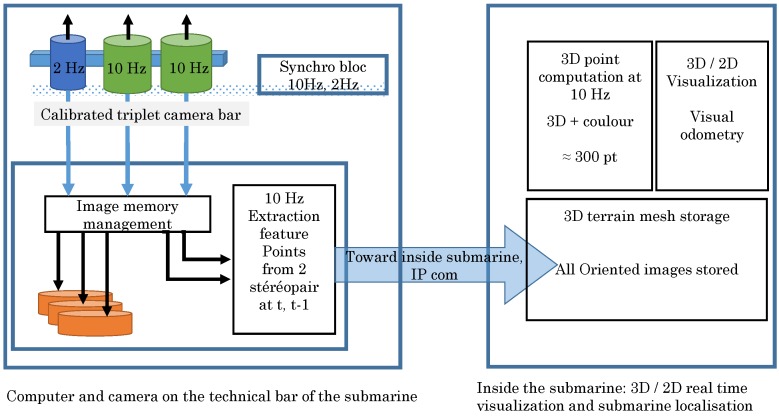
Synoptic description of the photogrammetric approach.

**Figure 6:**
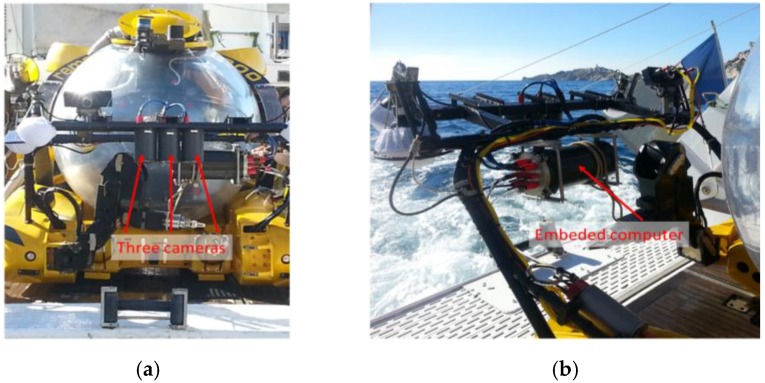
Images of the configuration used installed on the submarine; the technical bar of the Rémora 2000, the three cameras in their vertical cylindrical housing; (**a**) front view; (**b**) back view showing the embedded computer.

To achieve these goals, the system architecture (see Figure 5) must be able to record a large quantity of data in a synchronous way while performing real-time computations. Within given hardware constraints we have developed a modular architecture on which the different tasks are distributed. The first module is dedicated to image acquisition (two low-resolution cameras and one high-resolution camera), which is an electronic synchronization mechanism which ensures that all shots are correctly lit (see Figure 6). This synchronization mechanism is also used to tag images with homogeneous timestamps and to make it possible to retrieve image pairs (for real-time processing) and image triples (for metric high resolution processing). The three cameras are linked by an Ethernet/IP link to an on-board computer that store all produced images (see Figure 6b). Cameras, synchronization and storage can be configured and controlled by remotely sending UDP commands to the onboard computer.

Visual odometry computation is divided in two modules. The first module takes place on the on-board computer, it is responsible of extracting and matching feature points from the low resolution image pairs in real-time before they are stored. The extracted 2D homologous points are then sent through the Ethernet—TCP/IP link to the on-board computer were a second module is dedicated to the visual odometry calculation and visualization. The system presented here is patented by COMEX and the French National Centre for Scientific Research CNRS [8].

### 2.2. Photogrammetry

To ensure the complete coverage of the study area by the submarine, knowing its position in real-time is crucial. Since rigid transformation links the vehicle and the photogrammetry system, tracking the latter is sufficient for being able to deduce that of the submarine.

The motion of the photogrammetry system, consisting of three high-resolution cameras whose internal and external orientations are theoretically known, must therefore be evaluated. Its assembly should be calibrated before starting the mission. During the mission, the ego-motion of the system is computed on the fly via the embedded computer.

In the literature, the bundle adjustment methods used to refine the pose estimation have proved their effectiveness [9,10,11] and more generally the multiple views approach [12,13]. Nonetheless, a good approximation of initial values passed as input to the bundle adjustment method is required in order to speed up the convergence.

### 2.3. Orientation of Images

Let’s consider that the camera assembly to be oriented, where M_j_ is the set of projection matrices, observes a set of points *X_i_* in space and that *x_ij_* is the 2D projected point of the i^th^ 3D point on the j^th^ image frame. Therefore, the orientation of the images depends on finding values *M_j_* and *X_i_* that solve the following equation:

(1) $$M_{j}X_{i}\;–\;x_{ij}\;=\;0$$

here the matrix *M_j_* embeds both the rotation and the translation information. The bundle adjustment method proceeds to refine *M_j_* based on forming the Equation (1) as a minimization problem:

(2) $$\underset{M_{j}X_{i}}{min}\underset{i,j}{\sum }d\left(M_{j}X_{i},x_{ij}\right)$$

where *d(x,y)* is the Euclidean distance between two image points *x* and *y*. The minimization Equation (2) can be solved iteratively using the following convergence form:

(3) $$J^{T}J\delta =J^{T}\epsilon$$

where *J* is the Jacobian of the reprojection function (for more information, refer to [9,14]).

Using least squares approach is very costly in terms of computation resources in the given context. Moreover, its cost increases considerably as the number of parameters, *i.e.*, extrinsic parameters of the cameras and the 3D positions of the points, grows, so in order to reduce the calculation time, we have to reduce the number of parameters.

**Figure 7:**
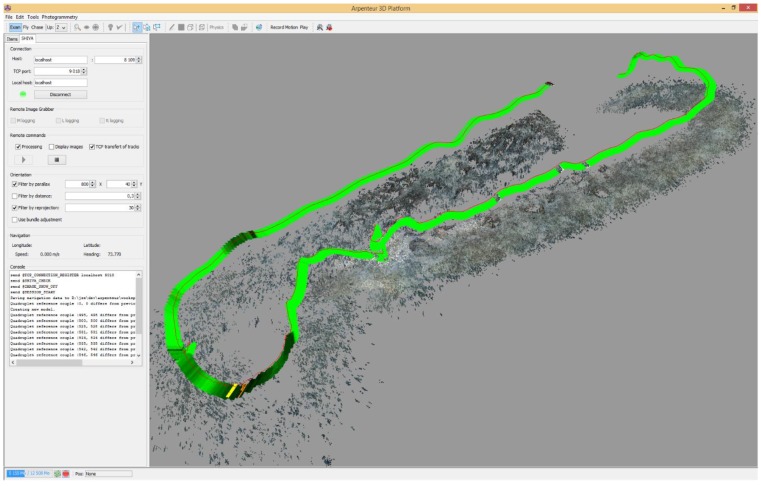
Real-time visual odometry as seen from the on-board computer. This figure shows the 3D point cloud calculated over the surveyed zone and the position of the vehicle for each image pair. The density of the points measured on each image is colour-coded (yellow, green and black) which gives an overview of the quality of the orientation (yellow < 30 points, green between 100 and 200 points, dark green > 300 points).

In our application, the photogrammetry system used is a stereo system whose relative pose is fixed and previously determined through a calibration phase. This characteristic allows to reduce the number of parameters linked to the cameras by a factor of 2 (see Xue and Su [15]). In fact, for a stereo pair, the extrinsic parameters of the right camera can be determined using those of the left camera. By taking this fact into account, Xue and Su proposed a method for bundle adjustment that reduces the number of parameters while keeping the information from the observations of the left and right images of the stereo pairs. This reduces the bundle adjustment computation time. The obtained results prove to be sufficient for our application. An illustration of the orientation of several images is shown in Figure 7.

### 2.4. Visual Odometry: Rapid Calculation of the Orientation of Images

In the context of relative motion estimation in robotics, Sünderhauf and his team [16] proposed a method that simplifies and accelerates the bundle adjustment. This method consists of working with a window containing a subset of consecutive images instead of all the images. The images in this window are oriented, and the estimated parameters are used for the initialisation of the next orientation, whose window is shifted one image further. Moreover, to compensate for the lack of precision due to small baseline distances of the images taken within the same window, only images whose positions are sufficiently separated (*i.e.*, using a preset threshold distance), are retained. This corresponds to the minimal distance of displacement of the vehicle between two images.

By combining the work of Sünderhauf and that of Xue and Su, we implemented a new algorithm in order to find the orientation of the stereo images taken by the submarine:

- Starting with image I_i_, we look for the closest image I_i+k_ so that the displacement between I_i_ and I_i+k_ exceeds a certain threshold. This threshold is experimentally around one centimeter for a camera shooting at a frequency of 10 Hz. This is determined using odometry.
- Add image I_i+k_ to the window.
- i becomes i + k and we repeat Step 1 until we have three stereo image pairs in the window.
- Apply the bundle adjustment method proposed by Xue and Su using the frames selected in the first step for the initialisation.
- Then, shift the window over one image and repeat the procedure until all images are processed.

### 2.5. Calculation of the Approximate Orientation of Images for Bundle Adjustment

In order to calculate the bundle adjustment as described in the previous paragraph, it is necessary to calculate the approximate position of the cameras. We have at our disposal a series of 2D points, homologous over four images (two consecutive pairs). The points correspondences have been established on the stereo camera pair at time t and the same at time *t − 1*.

At each step, a set of 2D points, which corresponds to pairs *t* and *t − 1* are sent to the on-board computer in order to calculate the relative position of the vehicle. The procedure of calculating the orientation of an image pair at time t with respect to an image pair at time *t − 1* is as follows:

- The vehicle moves slowly and we consider that the distance travelled between time *t* and *t − 1* is slight as well as the change in orientation. Under these conditions, we consider the camera exposures at time *t* are the same as camera exposures at time *t − 1*. Which is a good approximation due to the slow motion and the high image acquisition rate. Formally:
  (4) (RT)_right(t)_ = (RT)_right(t-1)_
  (5) (RT)_left(t)_ = (RT)_left(t-1)_
- Knowing the relative orientations of the left and right cameras and knowing that these values remain fixed in time, we can obtain the 3D points from the 2D points through triangulation, one time by using the image pair *t* and another by using the pair *t − 1*. We thus obtain two homologous point clouds calculated at time t and at time *t − 1* but with the camera exposures for times *t* and *t − 1*.

If the vehicle was effectively motionless, the two point clouds would be mixed together. In fact, the vehicle’s motion causes a displacement of the images which leads to a displacement of the point cloud that corresponds to the image pair *t − 1* with respect to such that corresponds to the image pair *t*. The rigid transformation [RT] required for expressing the cameras t in the reference pair *t − 1* is the rigid transformation required to move the 3D point cloud at time *t − 1* to the one obtained at time *t*. Hence, the problem of calculating the orientation of the cameras at time t in relation to time *t − 1* leads back to the calculation of the transformation used to move from one point cloud to the other. This is possible under our configuration, with small rotation.

Below, we present the method to compute the transformation for passing from the point cloud calculated at time t, denoted P, to the one calculated at time *t − 1*, denoted P’. So we have two sets of n homologous points P = {Pi} and P’ = {P’i } where 1≤i≤n. n is the size of the point cloud.

We have:

(6) $${{\text{P}}^{\prime }}_{\text{i}}=\text{R}\times {\text{P}}_{\text{i}}+\text{T}$$

where R is the rotation matrix and T is the translation.

The best transformation minimises the error err, the sum of the squares of the residuals:

(7) $$\text{err}=\overset{\text{n}}{\underset{\text{i}=1}{\sum }}∥{\text{RP}}_{\text{i}}+\text{T}-{{\text{P}}^{\prime }}_{\text{i}}{∥}^{2}$$

To solve this problem, we use the singular value decomposition (SVD) of the covariance matrix C, which shows to be robust and have low computation time. We note COMp and COMp′ the Centre of Mass of the set of 3D points P and P′:

(8) $$\begin{array}{c} \text{C}=\overset{\text{n}}{\underset{\text{i}=1}{\sum }}({\text{P}}_{\text{i}}-{\text{COM}}_{\text{P}})\times {\left({{\text{P}}^{\prime }}_{\text{i}}-{\text{COM}}_{{\text{P}}^{\prime }}\right)}^{\text{T}}\\ \left[\text{U},\text{S},\text{V}\right]=\text{SVD}\left(\text{C}\right)\\ \text{R}={\text{VU}}^{\text{T}} \end{array}$$

(9) $$\text{T}=-\text{R}\times {\text{COM}}_{\text{P}}+{\text{COM}}_{{\text{P}}^{\prime }}$$

Once the image pair tare expressed in the reference system of the image pair *t − 1*, the 3D points can be recalculated using the four observations that we have for each point.

A set of verifications are then performed to minimize the pairing errors (verification of the epipolar line, the consistency of the y-parallax, and re-projection residues).

**Figure 8:**
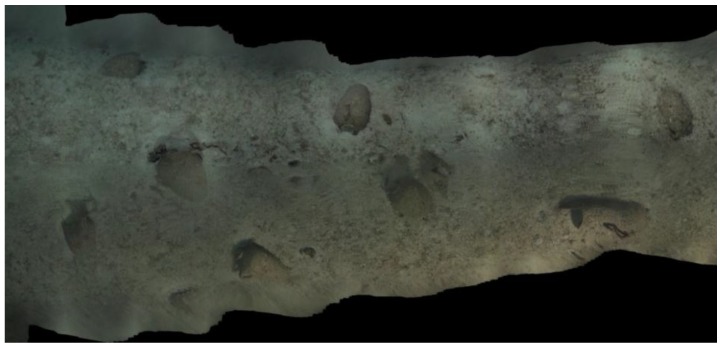
Partial orthophograph produced by the real-time visual odometry orientation. Some small imperfection are visible due to the orientation accuracy, however, the 3D model is visually appealing and acceptable to validate the survey.

Once validated, the approximated camera exposures at time t are used as input values for the bundle adjustment as described in the previous subsection. An example of reconstructed 3D model based on this method is shown in Figure 8.

### 2.6. Dense 3D Reconstruction

Following the orientation stage, in our case the orientation of two consecutive stereo pairs, the 3D point cloud that we obtained have low density. In the processing chain implemented here, the orientation of these two pairs is performed in a real-time loop at 10 Hz. It may be useful for operation managers to have a partial 3D model, for localization purposes, while the vehicle performs its survey. To do this, we developed a point cloud densification model based on the image sequence, which is possible as soon as the images are transferred on board (in the future, this densification could be performed using the embedded computer, but the current lack of resources of this machine makes it difficult. In fact, the embedded computer is subject to size, power consumption and temperature restrictions which affect its performance).

This densification is however necessary in order to reconstruct a realistic 3D model. This is done using Multi View Stereo (MVS) methods that produce a dense point cloud using images and the camera parameters. Furukawa [17] proposed a method based on a “patch” based reconstruction (Patch-Based Multi View Stereo or PMVS). They made a model of the surface S, with a random 3D point p from somewhere in the scene, and modelled, using a square section of a plane tangent to S at p, the patch. The 3D position of the patch is determined by minimizing the variance between these projections on the images. The algorithm functions in three steps:

- Initialization of a set of patches by interest points.
- Expansion, which consists of reconstructing new patches around those already identified.
- Filtering to strengthen consistency and remove any erroneous patches.

**Figure 9:**
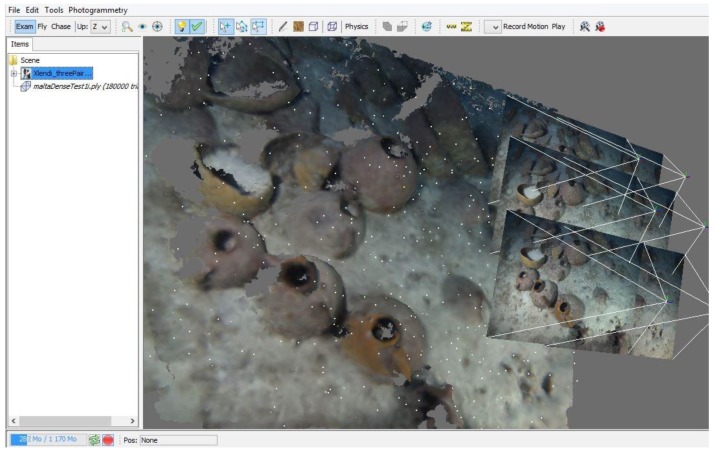
Three consecutive stereo pairs, oriented using the bundle adjustment approach described above, with a local densification of the original images. The visualisation is done inside the 3D tool developed by LSIS lab as a part of the Arpenteur project.

We have integrated this method in our processing chain (see Figure 9). On the other hand, contrary to PMVS, our developments directly use the images produced by the cameras, without any distortion correction nor rectification, with an adapted algorithm, dedicated to the calculation of epipolar lines.

**Figure 10:**
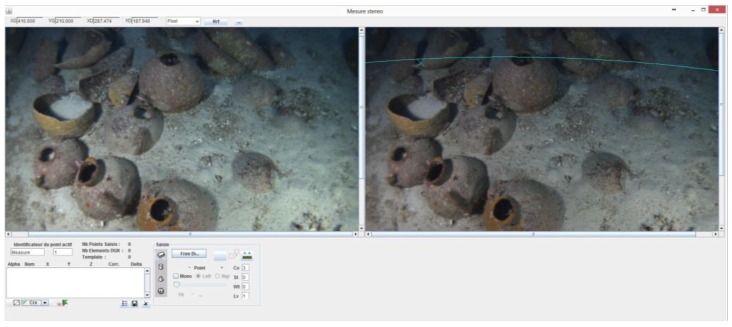
Photogrammetric stereo images taken during odometric navigation while surveying the Xlendi wreck. In the image on the right, one may observe a representation of the epipolar curve clearly showing the strong distortion present in images taken using underwater cameras. Modelling the epipolar line as an arc of circle is essential in calculating the visual odometry in real time as well as in the point cloud densification step (Photograph: GROPLAN consortium, LSIS, COMEX).

In fact, we applied a distortion transformation approach which made it possible to control the final adjustments and the precision of the resulting model. The distortion transformation proceeds by approximating the distorted epipolar line as a curve to simplify the calculation and this curve is modelled by an arc of circle based on the fact that the radial distortion is much greater. It mainly disturbs the projection of the scene on the image (see Figure 10). The following algorithm is used to calculate the equation of the epipolar curve for a point M:

(a) The M coordinates are corrected for distortion and eccentricity. Let $M^{\prime }$ denotes the corrected coordinates.
(b) Using the new coordinates, the equation for the ideal epipolar line l is determined as $l=FM^{\prime }$ where F where the Fundamental matrix is described in [13]. It is computed using the fixed calibration parameters.
(c) The two intersection points of l with the window of the image are calculated.
(d) The centre of mass of these two points is calculated.
(e) The distortion and eccentricity of Camera 2 are applied to the three points (the two intersection points and their centre of mass).
(f) The three new points thus obtained are the points that define the arc of circle.

### 2.7. Precision and Control

The assumption of having small baseline distances when computing the orientation in real-time, cannot give excellent results for the entire set of images. In fact, the main problem is limiting common points to only four or six images, *i.e.*, two or three consecutive stereo pairs. Although the visual odometry method presented here is sufficient to ensure the complete coverage of the site in real time, it does not provide enough precision for the final model, especially for our goal which is the automatic recognition of each artefact, and to evaluate its variance with the theoretical model.

We therefore implemented a second step where the homologous points are extracted and matched for all images as well as a global bundle adjustment was performed to ensure the best possible orientation. This was done whilst taking into account the constraints related to the set of three fixed and calibrated cameras.

Two software programs were then used to interface with our system: Agisoft’s PhotoScan and Bingo. The use of both programs permitted the control the final adjustments and the precision of the resulting model.

When comparing the results of the two models, the one obtained using odometry and traditional bundle adjustment which take into account all the possible observations of the 3D points, reveals the presence of residues of approximately 5 mm on the (X,Y) plane (which is lateral to the motion), and within 1cm range depth-wise (cameras pointing direction) on sequences with more than 1000 images. In fact, these data vary in function of the quality of the surveyed terrain. When the seafloor is sandy and low textured, the matched points are less and of lower quality. In the areas where the amphorae were found, the number of detected feature points is more, their quality is better, and the residues between models is less pronounced.

The overall 3D model, obtained using the global bundle adjustment applied on all the high-resolution images is scaled by introducing a stereo base of 0.297 m (value obtained after the triplet calibration, done before the mission in shallow water) as a constraint in the bundle adjustment. At the end, more than 1000 stereo-pairs poses were refined by this constraint so that the residues become less than one millimetre.

## 3. Ontologies, 3D Pattern Recognition and Modeling Artefact

One of the primary objectives of the GROPLAN project is to provide archaeologists with a set of measurement tools that do not require the presence of a specialist to be used. The goal is to obtain a 3D model of a site with archaeological information already integrated.

The development of such tools depends on the collaboration between experts from various fields of research along with measurement specialists. The transfer of knowledge between all the players involved requires the development of an appropriate knowledge representation.

We opted for a representation based on the notion of an archaeological entity, a notion already used in the Arpenteur project [18,19]. The basic structure is therefore an object-type structure, based on a concept taxonomy describing the archaeological knowledge involved as well as the photogrammetrical knowledge used for the survey. A double formalism is used for the implementation, Java programming language for computing and generating the survey from the images and OWL2 for its implementation by ontology, which is used to manage the coherence of the results. This double implementation allows for an effective procedural attachment and ensures the full use of the two aspects of this double implementation, logical and computational.

### 3.1. Multidisciplinary Knowledge

The development of measurement tools designed for use by non-specialists revolves around two axes:

- Understanding the needs of the experts in the field.
- Developing measurement methods that meet these needs.

An answer to the problem raised by these two axes is the development of concepts to represent objects that group together the expectations of the experts and that can be measured. These objects can be physical objects or sets of data required by experts for their work and which can be determined during the measurement process.

Starting from this informal description, we can reduce our notion of the field of knowledge to the knowledge-base linked to the objects. For example, in the scope of underwater archaeology, the field of knowledge consists of models of archaeological objects (amphorae, ships’ stores and wreckage) that include, among other aspects, metrological values, coherence relationships, dating information, as well as subsequent bibliographical information.

Once the field of knowledge notion is adapted to our needs, we can adjust to the understanding of the expert. We consider an expert in a given field as someone who acts as an interface between a field and others outside of the field. A field can have of course several experts and photogrammetrists themselves are, in the context of such collaboration, to be considered as experts.

The creation of a measurement system based on knowledge requires the collaboration of at least one expert in a field with one expert in measurements. This collaboration is only possible if knowledge representation is formalised and represents the required knowledge coming from the various fields defined.

### 3.2. Representing Objects in a Given Field

**Figure 11:**
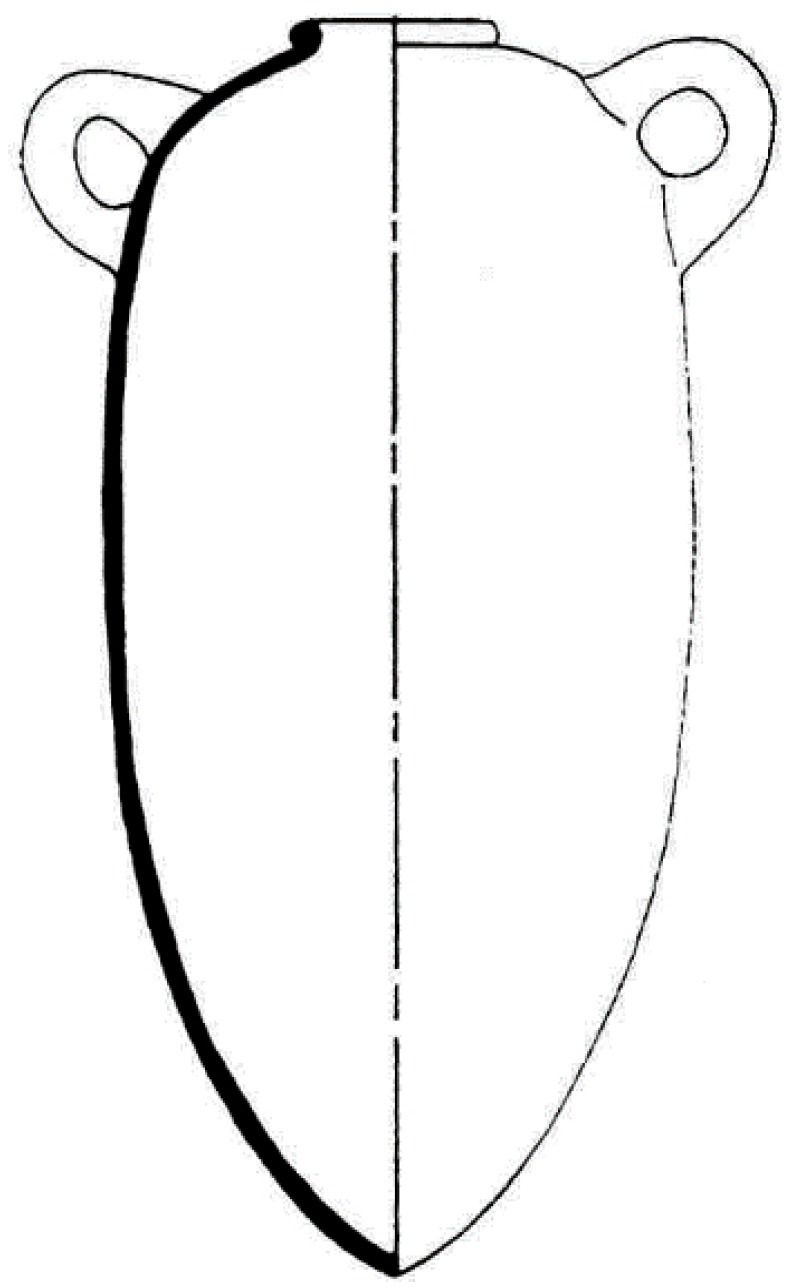
Standardised view of an amphora of type Ramon 2.1.1.1-72. [20].

The definition of objects in a given field is based on their formal description. Experts of a given field have a comprehensive knowledge of the precise descriptions of these objects based on heterogeneous information. In fact, this information can be in the form of data sheets, schematics or drawings, spatial data, textual information or literature, bibliographies, geographical information or even classifications. Figure 11 shows one type of data related to the description of amphora: their profile. In the specific case of amphora, the following information is available:

- metrological data (height, maximum diameter, volume, …);
- spatial data (position, convex envelope, 3D representation);
- physio-chemical data (type of pottery, colour, container analysis);
- archaeological documentation (chronologies, bibliographies, studies).

The media holding this information varies from data sheets filled in by archaeologists, to digital 3D models, as well as electronic field databases. Due to the heterogeneity of the available information and media, a formalism adapted to our problem is a conceptual formalism. Indeed, the definition of concepts is left up to relevant experts and different concepts are assembled into global concepts from a common knowledge viewpoint.

Formalized concepts are developed based on the knowledge from the field of research. The used descriptions as a basis for conceptualisation are expressed by publications, interviews with experts of the given fields, and pre-existing formalisms. In the scope of underwater archaeology for example, conceptualisation is based on the work of archaeologists such as Dressel or Ramon for certain amphorae discovered in the Phoenician wreck and more specifically the work concerning the identification of typologies is done by archaeologists more involved in the specific study of the wreck. Here, the Phoenician shipwreck is studied by Gambin and Sourisseau.

The first step to represent objects in a given field begins with a conceptualisation. Staring with heterogeneous descriptions, experts express an archaeological concept as well as set of relationships linking it to various other concepts that describe, for example, materials or a shape. This set of relationships and concepts can be used to understand the concept of an amphora. This can be then used during the measurement process as well as during the specific study carried out by the experts.

### 3.3. Notion of a Measurable Item

The conceptual representation of objects from different fields allows us to give expression to objects, or at least a portion of our knowledge of the object, from one field to another. However, it is impossible to develop measurement techniques and adapted tools if each set of concepts from a particular field is not intelligible to experts of another field. In the scope of this work, beyond our study of a cargo of amphorae, the implicated fields of study are varied and often independent or only possess slight common knowledge. For example, the study of the ship’s structure and its cargo can be carried out by different specialists each having their own specific knowledge. The construction of a representation that can be used by experts from different fields then depends on the establishment of a minimal body of knowledge that is coherent and shared by all experts and which also guarantees the transversality of the concepts employed by using a point of view shared by all. As the context of our work is based on photogrammetrical measurements, all the objects studied are necessarily measurable by photogrammetry. The concepts that characterize the items from all the fields involved (archaeology, architecture, biology) can then be expressed from a measurement point of view and share the notion of a measurable item. We define a “Measurable Item” as an item on which it is possible to carry out measurements. All the experts from any field can then extend the notion of a Measurable Item so that it can be further specified and integrate knowledge from a particular field. In addition to the in-site measurements, the surveyed items are intended to be studied and preserved in a museum. This aspect constraints the use of a dedicated ontology such as CIDOC-CRM. A strong link between the various ontologies is therefore necessary.

### 3.4. Taxonomy of Measurable Items

As mentioned earlier, all the concepts that characterize the items to be measured are sub-concepts of the measurable item. We can then organise the concepts from a measurement point of view by defining a set of relationships linked to their morphology and based on the information obtained during the measurement process. For example, an amphora can be characterized dimensionally by its maximum diameter, height and the internal and external diameters of its neck, the presence of a “shoulder”, as well as the ratio of its maximum diameter and its height. Some of these relationships are specific to amphorae and are used to define their typology, whereas others are common to certain concepts which subsume them either directly or in a more distant manner.

The presence of common relationships allows the concepts to be organised according to a heritage relationship. Each concept B possessing all the morphological relationships of concept A and having additional morphological relationships is a sub-concept of A. In this case, concept A is known as a super-concept, by analogy with the nomenclature of the Item Model. This heritage relationship allows us to define a taxonomy of measurable items.

### 3.5. Limits of the Taxonomy and Typology

The implemented taxonomy is expressed from a measurement point of view. Although it enables us to represent items coming from various fields in a single framework, it does not permit the specification of items beyond a certain level. In the field of underwater archaeology for example, amphorae are classified according to various typologies, such as for example, Ramon T.2.1.1.1 and Ramon T.2.1.1.2. It is impossible to represent these typologies as concepts of our taxonomy as all amphorae possess the same morphological attributes. The differentiating criterion between amphorae is not the existence of certain attributes, but rather their value, or even the relationship between these values. For example, the relationship between the height and its maximum diameter, or the height (Z side) where the amphora’s maximum diameter is located. Our taxonomy is unable to express such a classification because the critical criteria are completely linked to the field of study and their integration is incompatible with the hierarchical relationships that we use. In order to solve this problem, we defined the notion of typology. The typology of a measurable item is a set of value range and default values for the attributes of an item in its field of study. A typology is not a concept in the sense of our taxonomy, but is used to characterize an item using its links with the field of study involved. Typology can be seen as a parameter of the instantiation of the item that specifies the default values of its attributes. These values are set based on the knowledge from the field of study.

The typology of an item is based on the notion of a default morphological relationships and value ranges. It is possible to represent the value ranges using constraints on these relationships. An item is then characterized by a typology only if its morphological relationships are within the defined ranges. In order to verify the validity of the morphological relationship of an item for which we possess a typology, we must define constraints known as intrinsic constraints. For an item to be considered intrinsically coherent, it must meet all the intrinsic constraints related to it.

Just as items are organized into taxonomies, intrinsic constraints are too. An item from a concept C sub-concept of A must meet the intrinsic constraints related to C, but also those related to A if they exist. For example, a basic and general intrinsic constraint for all measured items: “The length of a measurable item is positive”. For every “measurable” item, this constraint must be met.

### 3.6. Completeness of Objects

By their nature, photogrammetrical surveys are incomplete. Since they are performed without any contact, in order to correctly record the artefacts’ position upon their discovery only the visible side of the objects lying on the sediment will be measured. The problem with the survey as presented here involves the confrontation of a measured object with its theoretical model. Two types of problems have to be resolved: The first is the incompleteness of the survey in regard to a theoretical model; the second is obtaining a complete theoretical model.

Indeed, the mass production techniques used at that era may encourage us to bring up the issue of an almost industrial constant theoretical model. However, the development of this model still depends on the choice of a paradigm instance. No theoretical blueprints can be of course consulted. Thus, archaeologists specialised in developing typologies publish blueprints and schematics of these typologies based on surveys of studied and compared instances. Furthermore, it should be also noted that a shipwreck of this importance holds a relatively large number of amphorae compared to that of amphorae of this type already identified in the world.

**Figure 12:**
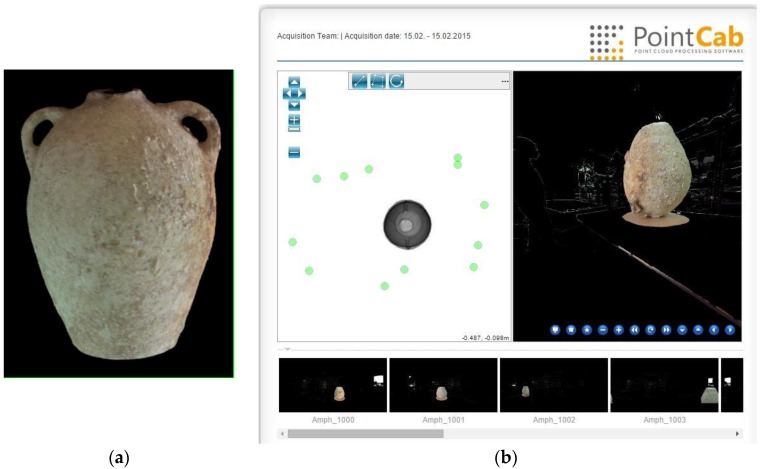
An exemplar output of a laser scanner of an amphora removed from wreck during a dig in July 2014; (**a**) a complete 3D model of an amphora; (**b**) the fusion of various captures of the laser scanner in order to obtain the complete 3D model (performed by DeMicoli & Associates with the University of Malta).

The development of a theoretical model is therefore not trivial and it must be validated for this shipwreck when all the visible amphorae have been recognised and modelled. In order to obtain a reliable theoretical model, we started with exhaustive surveys performed using a laser scanner system for few amphorae carefully removed from the site. An example of the scanning procedure is shown in Figure 12.

In this way we can obtain 3D models for certain amphorae coming from the wreck. Some other models have been already defined in bibliographical references. The confrontation of partial measurements of the amphorae or fragments present on the Xlendi wreck will take into account the origin of the theoretical model with the help of a variability threshold present in the measurements instantiation phase.

### 3.7. Knowledge Representation in the GROPLAN Project

Knowledge representation within the project is an extension of the conceptual representation. Two distinct aspects of the representation must be taken into account:

- the intrinsic aspect
- the extrinsic aspect

The intrinsic aspect defines the studied items. It consists of all the concepts with their relationships to heritage, characterisation and aggregation as well as the constraints concerning the default values and attributes. In fact, the conceptual representation is used to describe the properties of the entities studied, but does not give them a priori values or even limit the values they may have. In the scope of measurements based on knowledge, many properties can have default values or may even not possess certain values. Formally, an Entity is a set expressed as $\text{E}=\left\{\text{C},{\text{V}}_{\text{d}},{\text{C}}^{\text{I}},\text{R},{\text{C}}^{\text{E}}\right\}$ , where:

(1) C is a concept.
(2) V_d_ is a set of default values for its attributes.
(3) C_I_ is a set of constraints on its attributes.
(4) R is a set of relationships between instances of C.
(5) C_E_ is a set of constraints on its relationships of R.

Each item characterized by an entity E is an instance of class C. Components 1, 2 and 3 form the intrinsic part of the entity only apply to one item at a time. While the components 4 and 5 only apply to sets of items; they form the extrinsic part of the entity. The correspondence between a set of items O and an entity is called characterisation. In the scope of the study of amphorae measured by photogrammetry, the entities studied are based on the amphora concept that groups together attributes such as height, rim diameter, body diameter, *etc.*

In addition to the classic conceptual model, the notion of entity is based on the definition of constraints on the attributes. From a formal point of view, an intrinsic constraint applies to one or more attributes in order to set the boundaries of its values (for a numerical attribute), to specify a set of possible values (for a descriptive attribute) or even link values with different attributes.

In the scope of archaeology and more specifically the scope of measuring archaeological items such as amphorae, we can describe the intrinsic constraints based on knowledge compiled in the typologies and in the corpuses already measured. For example, the amphora Ramon 2.1.1.1, described in the work of Ramon [20], have a height of HR2111 ± Δh cm and a maximum diameter of DR2111 ± Δd cm. Of course, it is obvious that these constraints are necessary, but insufficient. The values HR2111, DR2111, Δh, Δd fall under archaeological expertise and are generally determined based on existing literature.

An entity characterizes a set O only if the following elements are verified:

- all the items o_i_ of O are instances of class C;
- the instantiation process to initialize the attributes of o_i_ with the values of V_d_;
- each item o_i_ of O satisfies the set of intrinsic constraints c_Ik_ of C_I_;
- all the constraints on the relationships c_El_ of C_E_ are satisfied.

Implementing the characterisation of a set of items by an entity means being able to enter values for the attributes of the concepts, to calculate the existence of relationships between the various items as well as evaluate the intrinsic and extrinsic constraints for the items studied.

## 4. Implementation in Java and Ontologies

**Figure 13:**
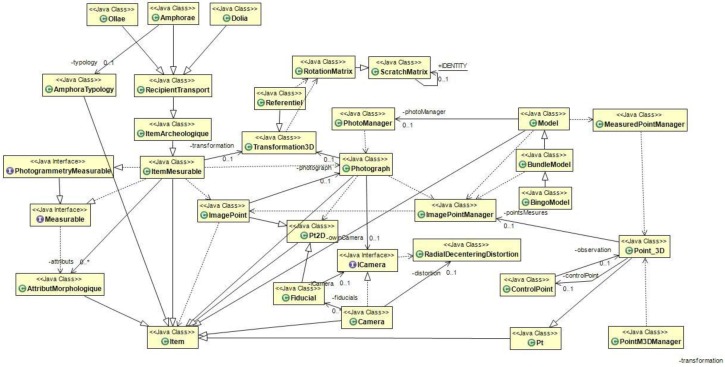
Partial UML diagram of Java classes managing the photogrammetrical process as well as the measured items.

The developments in Java are based on the ARPENTEUR platform [21], which includes various photogrammetric tools dedicated to heritage applications. It is designed to be used for photogrammetric measurements and the management of surveyed heritage artefacts. A taxonomy of measurable items is thus defined in agreement with specialists in the field and a photogrammetric measurement process is established for each item, hence, the knowledge of the field thus guides the photogrammetric measurements and ensures the consistency of the result (see the UML diagram in Figure 13). Although this approach is well structured from a software engineering point of view, it has been found to be limited with regards to its reasoning abilities concerning measured items as well as the weakness in representing inter- and intra-entity relationships. In fact, the problem in expressing these relationships rapidly became decisive, first when verifying the coherence of the instances in regards to their theoretical model and then the coherence of the organisation of the entities.

The proposed solution is a “double formalism”; the Java programming language for photogrammetrical computations and for measuring heritage artefacts, and the Web Ontology Language (OWL) in order to define an ontology that describes the concepts involved in the measurement process and the link with the measured items.

### 4.1. Implementation of the Representation by Entity Using OWL

For several years Web Ontology Language (OWL) has been used as a standard for implementing ontologies (W3C, 2004a). In its simplest form, it enables the representation of concepts (class), instances (individuals), attributes (data properties) and relationships (object properties).

The construction of an ontology in OWL, doubled with the Java taxonomy, is not done automatically. Each concept of the ontology is built so that it can be instantiated in Java but yet does not exactly reflect the Java tree structure. For example, the ScratchMatrix class in Java is inherited from the DenseMatrix class developed by Bjørn-Ove Heimsund in the framework of the Matrix Toolkit Java (MTJ) library. MTJ proposes a native implementation and a Java interface of the library Linear Algebra PACKage (LAPACK) originally written in Fortran, then used in MATLAB for solving systems of linear equations. You can see that the details of the Java implementation are not useful at the level of the ontological description. Nevertheless, it is essential to have the possibility of instantiating a Java matrix using OWL code and reciprocally being able to express an instance of the ScratchMatrix class in OWL (see the Java Figure 13 and OWL tree structures below).

For each concept of the ontology, a procedural attachment method was developed using JENA (an open-source Semantic Web framework for Java), each Java instance having a homologue in the ontology is capable of generating OWL content and possesses a constructor that can accept OWL contents as a parameter.

JENA is currently one of the most complete engines. It implements RDF, RDFS and OWL as well SPARQL queries. Moreover, a forward (Rete), backwards (logic programming) and hybrid chaining engine is available. This engine is used to implement the RDFS semantic and OWL.

Since Version 2 (W3C, 2009), OWL integrates the notion of constraints on the attributes (property restrictions) that are used to restrict possible values and their cardinalities. The OWL framework by itself enables the representation of a part of the entities (a concept C, its attributes, its set of relationships R and the sub-set of the intrinsic constraints restricting attributes cardinalities). The first step in the implementation of an entity concerns the concept itself, its attributes and the relationships it shares:

<owl:Class rdf:about=“#Amphorae”/>
<owl:Class rdf:about=“#AmphoraTypology”>
  <owl:equivalentClass><owl:Class>
      <owl:oneOf rdf:parseType=“Collection”>
        <rdf:Description rdf:about=“#Ramon_T2111”/>
        <rdf:Description rdf:about=“#Ramon_T2112”/>
      </owl:oneOf>
    </owl:Class></owl:equivalentClass>
</owl:Class>
<owl:DatatypeProperty rdf:about=“#hasHeight”>
  <rdfs:domain rdf:resource=“#Amphorae”/>

  <rdfs:range rdf:resource=“&xsd;double”/>
</owl:DatatypeProperty>
<owl:DatatypeProperty rdf:about=“#hasMaxDiameter”>
  <rdfs:domain rdf:resource=“#Amphorae”/>
  <rdfs:range rdf:resource=“&xsd;double”/>
</owl:DatatypeProperty>
<owl:ObjectProperty rdf:about=“#hasTypology”>
  <rdf:type rdf:resource=“&owl;FunctionalProperty”/>
  <rdfs:domain rdf:resource=“#Amphorae”/>
  <rdfs:range rdf:resource=“#AmphoraTypology”/>
</owl:ObjectProperty>
<owl:ObjectProperty rdf:about=“#isIntersectingBoundingBox”>
  <rdfs:range rdf:resource=“#Amphorae”/>
  <rdfs:domain rdf:resource=“#Amphorae”/>
</owl:ObjectProperty>


In this example, an amphora is represented by the concept Amphorae containing the attributes height (hasHeight), belly diameter (hasMaxDiameter) as well as a single typology (hasTypology). The various typologies of amphorae (limited here to Ramon T.2.1.1.1 and Ramon T.2.1.1.2) are represented by the listing AmphoraTypology. An amphora cannot be assigned dynamically to a given typology in OWL; in fact, this is determined by a set of constraints on the attributes that OWL cannot express.

Since 2004, the OWL framework was extended with the Semantic Web Rule Language (SWRL) (W3C, 2004b) that is able to define rules for classes and properties in order to deduct new information from a set of individuals as well as verify its coherence. Several OWL/SWRL inference engines, also known as semantic thinkers, are currently available and offer an acceptable level of performance for managing sets of individuals (Pellet, Hermit, RacerPro, *etc.*). Formally, an SWRL rule is defined as a Horn clause reduced to unary and binary predicates that express a datatype property, an object property or its belonging to a class. In the case of an amphora belonging to the typology Ramon T.2.1.1.1, we can write the following constraint on the metrology:

hasTypology(a, Ramon_T2112) ← Amphore(a) ^ sup(a.height, 90) ^ inf(a.height, 140)

This clause can be translated into SWRL as follows:

<swrl:Variable rdf:ID=“amphora”/> <swrl:Variable rdf:about=“#height”/>
<swrl:Imp rdf:about=“#Ramon_T2111-metrology”>
  <swrl:body rdf:parseType=“Collection”>
    <swrl:ClassAtom> 
      <swrl:classPredicate rdf:resource=“#Amphorae”/><swrl:argument1 rdf:resource=“#amphora” />
    </swrl:ClassAtom>
    <swrl:DatavaluedPropertyAtom>
      <swrl:propertyPredicate rdf:resource=“#hasHeight”/>
      <swrl:argument1 rdf:resource=“#amphorae”/><swrl:argument2 rdf:resource=“#height”/>
    </swrl:DatavaluedPropertyAtom>
    <swrl:BuiltinAtom>
      <swrl:builtin rdf:resource=“&swrlb;greaterThan”/>

      <swrl:arguments><rdf:List><rdf:first rdf:resource=“#height”/><rdf:rest>
        <rdf:List><rdf:first rdf:datatype=“&xsd;double”>90.0</rdf:first>
          <rdf:rest rdf:resource=“&rdf;nil”/>
          </rdf:List></rdf:rest>
        </rdf:List></swrl:arguments>
    </swrl:BuiltinAtom>
    <swrl:BuiltinAtom>
      <swrl:builtin rdf:resource=“&swrlb;lessThan”/>
      <swrl:arguments><rdf:List><rdf:first rdf:resource=“#height”/><rdf:rest>
        <rdf:List><rdf:first rdf:datatype=“&xsd;double”>140.0</rdf:first>
          <rdf:rest rdf:resource=“&rdf;nil”/>
          </rdf:List></rdf:rest>
        </rdf:List></swrl:arguments>
    </swrl:BuiltinAtom>
  </swrl:body>
  <swrl:head rdf:parseType=“Collection”>
      <swrl:IndividualPropertyAtom><swrl:propertyPredicate rdf:resource=“#hasTypology”/> 
        <swrl:argument1 rdf:resource=“#amphorae” />
        <swrl:argument2 rdf:resource=“#Ramon_T2111” />
      </swrl:IndividualPropertyAtom>
  </swrl:head>
</swrl:Imp>


The SWRL rule previously defined has two purposes. First, it assigns an amphora to a typology in function of its metrological attributes; second, it ensures the coherence of the information because if the amphora is already associated to a different typology, the inference will generate an incoherence by assigning it to the Ramon T.2.1.1.1 typology, which contradicts the typology uniqueness.

An entity is therefore completely implementable if limited to the use of OWL2 and SWRL. The table below shows the various OWL/SWRL components involved:
EntityOWLConceptowl:ClassInstanceowl:NamedIndividualAttributeowl:DatatypePropertyRelationshipowl:ObjectPropertyIntrinsic constraintowl:Restriction/SWRL ruleExtrinsic constraintSWRL rule

More formally, this representation is based on an OWL2/SWRL sub-set limited to its descriptive part (OWL-DL). The entity and its associated instances can therefore be implemented.

### 4.2. The Link with CIDOC-CRM

The ontology developed in the framework of the GROPLAN project takes into account the manufactured items surveyed, as well as the method used to measure them; in this case, photogrammetry. The surveyed item is therefore represented from the measurement point of view and has access to all the photogrammetrical data that contributed to its measurement in space. Two ontologies are aligned in this context; one dedicated to photogrammetrical measurement and the geo-localisation of the measured items, whereas the other is dedicated to the measured items, principally the archaeological artefacts, describing their dimensional properties, ratios between main dimensions, and default values.

These ontologies are developed with close links to the Java class data structure that manages the photogrammetric process as well as the measured items. Each concept or relationship in the ontology has a counterpart in Java (the opposite is not necessarily true). Moreover, surveyed items are also archaeological items studied and possibly managed by archaeologists or conservators in a museum. It is therefore important to be able to connect the knowledge acquired when measuring the item with the ontology designed to manage the associated archaeological knowledge. CIDOC CRM is a generic ontology that does not support the items that it represents from a photogrammetric point of view, a simple mapping would not be sufficient and an extension with new concepts and new relationships would be necessary.

This modelling work is based on a previous study that started from the premise that collections of measured items are marred by a lack of precision concerning their measurement, assumptions about their reconstruction, their age, and origin. It was therefore important to ensure the coherence of the measured items and potentially propose a possible revision. For more information, see [22,23,24,25,26].

The extension of the CIDOC-CRM ontology is structured around the concept E22 Man-Made Object. The root of ItemMesurable developed in GROPLAN extends this concept.

The mapping operation is done in Java by interpreting a set of data held by the Java classes as a current identification of the object: 3D bounding box, specific dimension such as maximum diameter or rim diameter in case of amphorae. These attributes are then computed in order to express the right CRM properties.

For example, the amphorae typology, which is strongly connected with the E52 Time-Span, in our point of view, the amphorae typology is linked with some relations between maximum height and maximum diameter (and of course others). This means that E52 instance of our amphora is filled in after a set of computations performed by the Java instance.

Several methodologies can be chosen regarding mapping two ontologies. For example, Amico and his team [27] choose to model the survey location with an activity (E7) in CRM. They also developed a formalism for the digital survey tool mapping the digital camera definition with (D7 Digital Machine Event). We see here that the mapping problem is close to an alignment problem which is really problematic in this case. Aligning two ontologies dealing with digital camera definition is not obvious; a simple observation of the lack of interoperability between photogrammetric software shows the wildness of the problem. We are currently working on an alignment/extension process with Sensor ML which is an ontology dedicated to sensors. Although some work have already been achieved [28,29], but not enough to clearly hold the close range photogrammetry process, from image measurement to artefact representation.

**Figure 14:**
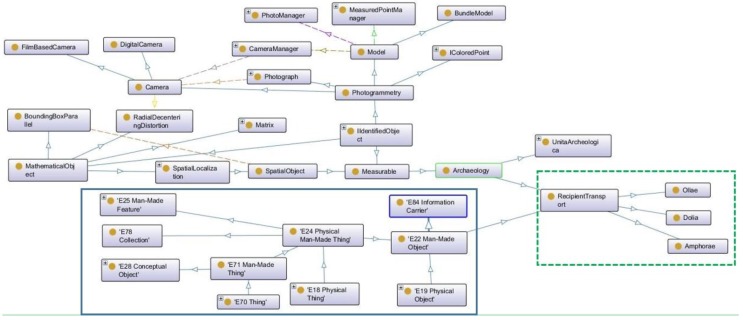
Partial view of the GROPLAN ontology and the link with CIDOC-CRM. (Screen shot taken from the “Protege” software application). In the blue frame, a sub-tree of the CIDOC-CRM ontology.

As the link between the data structure of the Java classes (Figure 13) and the GROPLAN ontology (Figure 14) is not trivial, the calculation of the properties of the individuals represented in the ontology is not a simple reproduction of the existing values in the corresponding Java instances. Let’s take a simple example: The unit in which the sizes of the measured items are expressed. For CIDOC-CRM, these sizes can be represented by the class E54 Dimension, which possesses a property P91 has unit. It is effectively rigorous to assign a unit to a size. Nonetheless in the scope of the Java class data structure representing the photogrammetrical process as well as the measured items; it’s not exactly the same thing. Photogrammetrical measurements use pixels for images. Once the images are oriented, this is done by the class Model (in the context of photogrammetry, a model is a set of images having common points and being oriented in the same reference system). 2D points observed in several images are calculated in 3D in the reference system of the model. It is therefore the model that contains the pixel/site reference transformation and with that the unit in which the 3D measurements are expressed.

Measured items do not have a reference to a property unit but towards a photogrammetrical model that contains a set of images in which this item was seen and measured, as well as a reference system defining the unit for expressing 3D measurements. Property P91 is therefore filled in indirectly by the Java instance that possesses a reference to the model that generated it.

### 4.3. 3D Object Recognition

3D shape retrieval is still an open field of research. Knowing that until now it is difficult to find an automatic method that is efficiently able to identify a 3D model with occlusion. Many existing 3D shape retrieval methods require a complete surface model of a query object in order to find it in the dataset [4,30,31,32] while others seek to find a signature that is invariant to rotation of the object [33,34,35]. We are mainly interested to partial shape retrieval methods and we refer the reader to a survey article by Yi *et al.* [36] for more details.

In this work, we present a novel object recognition approach that deals with partial objects. The purpose is to search for a partial object in a training dataset that contains full 3D objects (point clouds). Our algorithm is based on creating a dataset of partial 2D projections that are called level curves, whenever there is an enquiry, we search the dataset in order to find the correspondence. For creating the dataset, we take as input a set of 3D objects. We subsequently produce a set of samples of level curves by using a viewing sphere. The level curves are a set of 2D planar contours that are the projection of points on several perpendicular planes. The viewing sphere contains the target object and represents the base of samples on which the level curves are obtained. Our matching algorithm, used to compare level curves, is based on 2D planar curves alignment by using the intrinsic properties of curves which are the curvature and the arc-length. The properties are used by Sebastian *et al.* [37] for whole-to- whole matching curves.

#### 4.3.1. Related Work

There exist two main approaches that address the problem of partial shape retrieval; the local descriptors-based methods and the view-based methods. Local descriptors-based methods aim to extract the description (or signature) in the neighborhood of surface points whereas the methods based on view generate a set of 2D images of a 3D model from different points of view by projection. Partial shape retrieval ends to compare views. Johnson and Hebert [38] introduced the concept of spin images where they compute a 2D histogram of the 3D points projections on the cylindrical coordinates, Yi *et al.* [36] propose to use this signature with Monte-Carlo sampling on the surface model. Rusu *et al.* [39] propose Fast Point Feature Histograms (FPFH), an optimized method for real time use that characterizes the local geometry of a 3D point and stores the information in 16-bin histograms. Malassiotis *et al.* [40] extract a descriptor from snapshots of the surface over each point using a virtual camera oriented perpendicularly to the surface around the point. Cornea *et al.* [41] used the curve-skeleton of a 3D shape and compare between curves, by using Earth Mover’s Distance [42] to evaluate the partial similarity. Sun *et al.* [43] generate a sequence of 2D planar contour by projecting the geodesic circles onto the tangent plane. In this work, we introduced a new approach for partial 3D object retrieval from a database by using level curves. The originality of this work is to use a viewing sphere to extract the contours of the object from several viewpoints in order to create a database with complete information about the 3D objects. Next, the extraction of level curves that present the contours of the object at various levels in order to reduce the problem of matching between two 3D point clouds to a matching between 2D planar curves.

#### 4.3.2. The Approach

3D object models are used in many applications, such as computer vision, computer graphics and Computer-aided design. We can find a large number of databases of 3D models on the Web such as: Stanford 3D Scanning Repository [44], NTU 3D Model database [45] and 3D Keypoint Detection benchmark [46]. In this work, we used some 3D models from [44,46] and by using viewing sphere on each 3D model, we create our dataset where 3D models are represented by level curves obtained from each viewpoint. To find out if a partial model is part of an existing 3D model of our dataset, we have to match level curves of that partial model against all other in our dataset.

**Figure 15:**
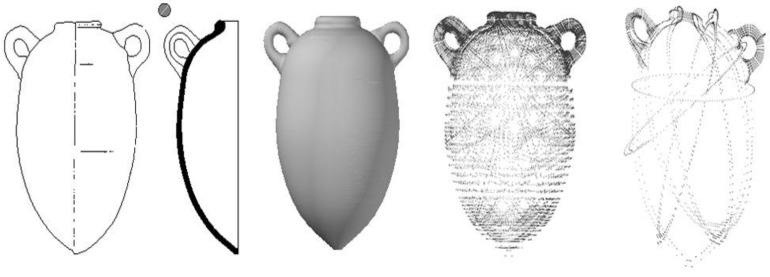
From left to right; the amphora model designed by the archaeologist, then the 3D model computed from the archaeological design. Two illustrations of level curves extraction using several planes.

As mentioned in the previous section, our approach is based on curve matching, the fact that we believe that the best description of 3D objects are their contours, led us to think of the level curves. Those curves can be extract by slicing out point clouds (3D models) using several planes with a regular step (see Figure 15). Two ways of slicing are possible: using one point of view *i.e.*, one “cutting” plane shifted along the model typically as level curve with horizontal plane in cartography. Or choosing the cutting direction from several point of view settable on a sphere defined around the studied object. Here we consider the latter case as it produce more complete information about the object, despite the larger number of curves that will be created which affects the computation time.

Matching process aims to extract the best part from one curve that matches as whole or just part of the second curve, to solve this problem, we need to find the position where the query curve aligns the best curve from the dataset. To measure the similarity between the query curve against the curves from the dataset, we slide the signature of the query curve on the current curve of the dataset. The small Euclidean distance indicates the position of the best fitting.

**Figure 16:**
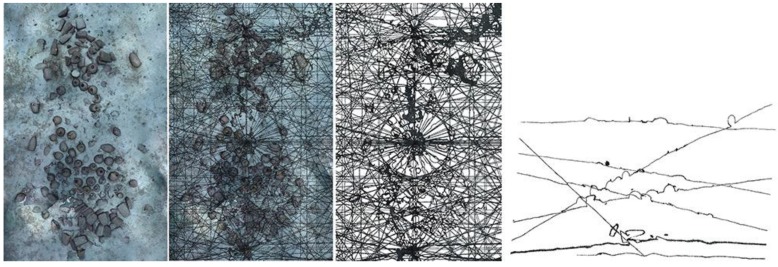
On the left, the ground 3D model computed by photogrammetry, on the right, level curves extraction from several planes with partial trace of the amphorae and other artefacts.

To remove the false matches, we added a new step to compute the Euclidean transformation between points on the matched parts. The alignment error is computed by using Root Means Square Error (RMSE), which represents the sum of distances among the points of matched parts. This error and the similarity measure can be used as indications on the quality of the curve matching. Figure 15 on the right shows an example of curve extraction from the complete 3D model of amphora Ramon 2111-73. This curve is matched with the illustrated curves in Figure 16 which shows the problem of the direction of parameterization as it is highlighted in [47]. Therefore, to solve this problem, each curve from the dataset is matched by using the first and second direction of the query curve parameterization, then the best match is kept.

#### 4.3.3. Experiments and Results

**Figure 17:**
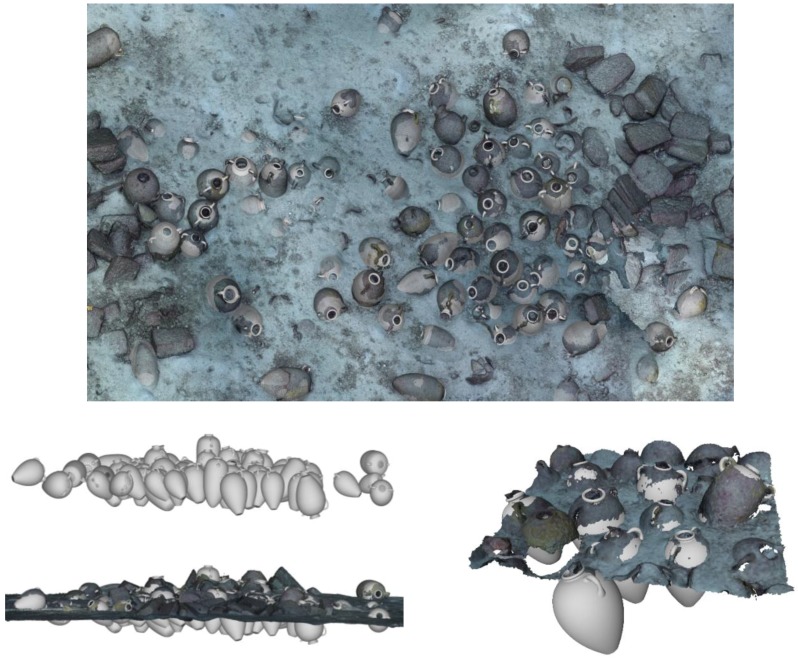
First matching using a manual recognition of the typology.

We first performed 3D partial object retrieval experiments using the publicly available database [44]. This work has already been published in [48]. In this paper, we work on a real case of the Phoenician wreck as shown in Figure 1 and Figure 3. Figure 4 shows the first step of this work in progress. Our target is to make our automatic matching algorithm reaches the accuracy of the manual matching (which is indeed an effort and time consuming task) as shown in Figure 17. Although the proposed approach is able to correctly detect the position of amphorae, rotation alignment is not accurate enough in some cases due to the small overlap. A possible solution is to extend matching aspects by considering other aspects such as colour and texture information.

The resulting 3D representation is very useful to archeologists as it furnishes a simple manner to interpret information about the localisation and position of amphorae. This facilitates any further exploration and inspection missions. Furthermore, it provides a fundamental platform upon which an accurate excavation strategy can be developed. Such an underlying strategy has direct impacts on aspects such as excavation techniques, conservation and budget.

**Figure 18:**
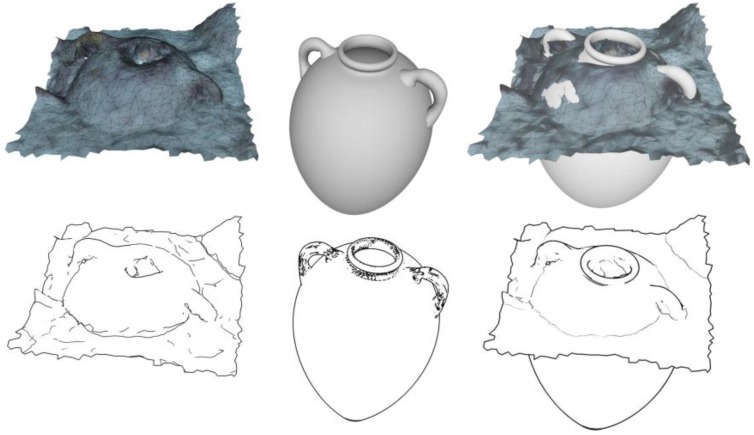
First row: Object recognition based on 3D models using point cloud representation. Second row: Illustration of object recognition using NPR representation.

On the other hand, when it comes to graphically representing an object of interest, a specialist in one domain may provide a different description of the object based on his own interest. This description may vary from one domain to another. In our context, what we perceive as easy to interpret and visually appealing, may not be the case for a researcher from another field. This is certainly the case with the 3D models produced. Other types of modelling can be further exploited to provide complementary information with respect to the given 3D point cloud-based representation. In this scope, we have achieved an attempt to generate Non-photorealistic rendering (NPR) of the scene. The 3D model obtained preserves the geometry of the original model, whereas the boundaries are highlighted. An example is given in Figure 18, here the model on the bottom right may bring more relevant information to the domain of archeology. Furthermore, the NPR representation can be used at the beginning to identify an area where an amphora is probably located. It can also be used during the process to improve object recognition and matching as an alternative to the current proposed matching scheme. The work presented here is still in progress and the first experiments are yielding very encouraging results. As shown in Figure 18, the highlighted object’s boundaries in NPR model represent a discriminative feature to recognise amphorae.

## 5. Ongoing and Future Developments

### 5.1. Pattern Recognition and Measurement

The goal of object recognition is to automatically and efficiently extract the relevant contents of a site; *i.e.*, determine the identity of the objects (of which a model is known a priori) present on the site as well as its spatial orientation.

In the scope of the GROPLAN project, we plan to integrate a pattern recognition tool for exploring underwater archaeological sites. This will help the archaeologist when studying the site, notably during the recognition and orientation phases. There are two types of available data, on one hand a set of models describing the items potentially present on the site, which is currently limited to amphorae, but this database can later be broadened by the user. And on the other hand, a 3D point cloud, measured on the site, obtained through an automatic photogrammetry process and whose density is roughly the same as that used to describe the models of the sought items.

The items present on the site are either damaged or partially covered with sediment; the objective is to help the archaeologist by determining the position and orientation of these items in space and suggesting a class to which they belong. For this aim, we are planning to exploit the 4PCS algorithm proposed by Aiger [49]. This allows the registration of two point clouds and deals with the problem of noisy data. The method is based on extracting all co-planar 4-points sets from a 3D point cloud, that are approximately congruent, under rigid transformation, to a given set of coplanar 4-points. To apply this algorithm in our context, a series of pre-processing steps have to be performed in order to prepare the two input point clouds to match the input requirements constrained by the algorithm in terms of noise and sufficient overlap. This approach has been already used in archaeological context by Reuter in the SeARCH project [50].

Furthermore, inspired by the works of Agarwal and Triggs [51] and Fei-Fei [52], we suggest modelling the appearance and the geometric configuration of the local areas of an item in a descriptor vector. The local areas will be represented by the element closest to a pre-calculated visual vocabulary and the geometric relationships between all the visual word pairs (elements of the visual vocabulary) detected in the model will be encoded in a descriptor vector. A classifier will be used to detect areas containing the item in the site. During the recognition phase, local areas will supply a certain number of matches and they will be used as a verification step to validate their similarity.

We plan to test several types of local descriptors such as Spin Image, 3D shape contexts as well as harmonic shape contexts [53]. In the learning phase, we will use SVM approaches where we will test several variations (SVM decision tree [54], multi-class SVM [55]).

### 5.2. Application on Nautical Archaeology

The first and most important aspect of photogrammetry on nautical archaeology is cost. Reducing time spent underwater and the amount of equipment deployed on an archaeological survey, independently of its depth, is a major requirement in the discipline. Likewise, so is the development of a theory of knowledge in nautical archaeology. The application of an ontology and a set of logical rules for the identification, definition, and classification of measurable objects is a promising methodology to assess, gather, classify, relate and analyze large sets of data, which also permit the development of broader studies related to the history of seafaring.

Nautical archaeology is a recent sub-discipline of archaeology, which developed after 1960. It is therefore normal that its early steps were concerned with recording methodology and accuracy in underwater environments. Techniques which permit working in specific conditions impose an array of practical constraints. These include reduced time to work and have less light, low visibility, a narrower field of vision, and other conditions such as those derived from surge, current, or depth [56,57,58]. Since the inception of nautical archaeology, theoretical studies aimed at identifying patterns and attempting to address larger anthropological questions related to culture change. However, the sample sizes were too small to allow generalizations. Few seafaring cultures have been studied and understood well enough to allow a deep understanding of their history, culture, and development. A good and perhaps unique example is the Scandinavian Vikings [59].

Through our work we hope to provide researchers from different marine sciences with the appropriate tools for the facilitation of their work that will in turn contribute to the aforementioned broader studies related to cultural changes.

The study of the history of seafaring is the study of the relations of humans with rivers, lakes, and seas, which started in the Palaeolithic. An understanding of this important part of our past entails the recovery, analysis, and publication of large amounts of data, mostly through non-intrusive surveys.

The methodology proposed in GROPLAN aims at simplifying the collection and analysis of archaeological data, and facilitating the establishment of relations between measurable objects and concepts. It builds upon the work of Steffy, who in the mid-1990s developed a database of shipbuilding information that tried to encompass a wide array of western shipbuilding traditions through time and relate conception and construction traits in a manner that allowed comparisons and the establishment of new relations between objects. Around a decade later Monroy transformed Steffy’s database into an ontological representation in RDF-OWL, and expanded its scope to potentially include other archaeological materials [10,60,61]. After establishing a preliminary ontology, completed through a number of interviews of naval and maritime archaeologists, Monroy combined the database with a multi-lingual glossary and built a series of relational links to textual evidence that helped contextualize the archaeological information contained in the database. His work proposed the development of a digital library that combined a body of texts on early modern shipbuilding technology, tools to analyse and tag illustrations, a multi-lingual glossary, and a set of informatics tools to query and retrieve data [10,60,61,62,63,64].

The GROPLAN approach extends these efforts into the collection of data, expands the analysis of measurable objects, and lays the base for the construction of extensive taxonomies of archaeological items. The applications of this theoretical approach are obvious, in that it simplifies the acquisition, analysis, storage, and sharing of data in a rigorous and logically supported framework. From a practical viewpoint, GROPLAN is also advancing the development of lighter, cheaper, and easier to handle equipment packages.

These two advantages are particularly relevant in the present political and economic world context brought about by globalization. The immediate future of naval and maritime archaeology depends on a paradigm change. Archaeology is no longer the activity of a few elected scholars with the means and the power to define their own publication agendas. The survival of the discipline depends more than ever on the public recognition of its social value. Cost, accuracy, reliability (for instance established through the sharing of primary data), and its relation with society’s values, memories and amnesias, are already influencing the amount of resources available for research in this area. GROPLAN stands as a pioneer and major contribution to the advancement of not only nautical archaeology, both in shallow and deep water, but its applications extend to land archaeology as well, and tie with the needs of a widening group of stake holders, which include a growing public.

As stated in the beginning of this paper, the main objective of this project is the development of an information system based on ontologies and capable of establishing a methodology to acquire, integrate, analyse, generate and share numeric contents and associated knowledge in a standard, homogenous form. Although still in an early stage of development, the GROPLAN approach has the potential to open a paradigm changing research direction. Even if we consider only questions related with the storage and sharing of primary data, GROPLAN has the potential to advance a set of basic rules of good practice in maritime archaeology. In 2001 the UNESCO Convention for the Underwater Cultural Heritage established the necessity of making all data available to the public [65]. GROPLAN project builds upon this philosophy, proposes a way to share archaeological data, and will undoubtedly change the rules in the field.
